# A Hint from America

**Published:** 1890-04-05

**Authors:** 


### A HINT FROM AMERICA.

It is everywhere difficult enough to raise money for charitable purposes. Hospital secretaries may therefore thank us for reproducing from the report of the St. Luke's Free Hospital, Chicago, "the ways by which the readers of that report can help the hospital." They are as follows:—

"I. You can support a bed by paying 300 dols. (£60) a-year in quarterly instalments.

"II. You can subscribe a certain sum monthly on the cards distributed in your church on Hospital Sunday, or to be procured from the Directress in your parish, who will collect your subscriptions.

"III. You can, among your acquaintances, collect funds and forward them to the treasurer, N. K. Fairbank, No. 19, Wabash Avenue.

"IV. You can organize Hospital Aid societies in your church and among the children of your Sunday school; by work or by subscription, obtain a large amount.

"V. You can send jellies, fruits, and delicacies from your table, or canned fruit, clothes, linen rags—in short, anything that is useful in a house.

"VI. If you live in the country, you can beg from farmers potatoes, butter, eggs, and vegetables of all kinds.

"VII. When you make your will, you can do as other kind people have done before you—remember the hospital by some bequest.

"VIII. You can speak a good word for the hospital when you are among strangers, and you can pray for it.

"IX. You can contribute something towards the endowment of beds. One is now in process of development."

It will be gratifying to know if any of our readers possessed of means and influence are induced to adopt any one of the above courses, with the view of helping the hospital or hospitals which they may know about or be interested in.

